# Detection rate for *ESR1* mutations is higher in circulating‐tumor‐cell‐derived genomic DNA than in paired plasma cell‐free DNA samples as revealed by ddPCR

**DOI:** 10.1002/1878-0261.13787

**Published:** 2025-01-04

**Authors:** Stavroula Smilkou, Aliki Ntzifa, Victoria Tserpeli, Ioanna Balgkouranidou, Alkistis Papatheodoridi, Evangelia Razis, Helena Linardou, Christos Papadimitriou, Amanda Psyrri, Flora Zagouri, Stylianos Kakolyris, Evi Lianidou

**Affiliations:** ^1^ Analysis of Circulating Tumor Cells Lab, Laboratory of Analytical Chemistry, Department of Chemistry University of Athens Greece; ^2^ Department of Medical Oncology University General Hospital of Alexandroupolis Greece; ^3^ Department of Clinical Therapeutics, School of Medicine, Alexandra Hospital National and Kapodistrian University of Athens Athens Greece; ^4^ Hygeia Hospital Athens Greece; ^5^ Oncology Unit Metropolitan Hospital Athens Greece; ^6^ Oncology Unit, Aretaieion University Hospital National and Kapodistrian University of Athens Greece; ^7^ Department of Medical Oncology, Second Department of Internal Medicine, “Attikon” University General Hospital, Athens Medical School National and Kapodistrian University of Athens Greece

**Keywords:** breast cancer, circulating tumor cells, circulating tumor DNA, droplet digital PCR, *ESR1* mutations, liquid biopsy, plasma cell‐free DNA

## Abstract

Plasma cell‐free DNA (cfDNA) analysis to track estrogen receptor 1 (*ESR1*) mutations is highly beneficial for the identification of tumor molecular dynamics and the improvement of personalized treatments for patients with metastatic breast cancer (MBC). Plasma‐cfDNA is, up to now, the most frequent liquid biopsy analyte used to evaluate *ESR1* mutational status. Circulating tumor cell (CTC) enumeration and molecular characterization analysis provides important clinical information in patients with MBC. In this study, we investigated whether analysis of CTCs and circulating tumor DNA (ctDNA) provide similar or complementary information for the analysis of *ESR1* mutations. We analyzed both plasma‐cfDNA (*n* = 90) and paired CTC‐derived genomic DNA (gDNA; *n* = 42) from 90 MBC patients for seven *ESR1* mutations. Eight out of 90 (8.9%) plasma‐cfDNA samples tested using the ddPLEX Mutation Detection Assay (Bio‐Rad, Hercules, CA, USA), were found positive for one *ESR1* mutation, whereas 11/42 (26.2%) CTC‐derived gDNA samples were found positive for at least one *ESR1* mutation. Direct comparison of paired samples (*n* = 42) revealed that the *ESR1* mutation rate was higher in CTC‐derived gDNA (11/42, 26.2%) than in plasma‐cfDNA (6/42, 14.3%) samples. Our results, using this highly sensitive ddPLEX assay, reveal a higher percentage of mutations in CTC‐derived gDNAs than in paired ctDNA in patients with MBC. CTC‐derived gDNA analysis should be further evaluated as an important and complementary tool to ctDNA for identifying patients with *ESR1* mutations and for guiding individualized therapy.

## Graphical abstract

Analysis of *ESR1* mutations in plasma cell‐free DNA (cfDNA) is highly important for the selection of treatment in patients with breast cancer. Using multiplex‐ddPCR and identical blood draws, we investigated whether circulating tumor cells (CTCs) and cfDNA provide similar or complementary information for *ESR1* mutations. Our results indicate a higher detection rate for *ESR1* mutations in CTCs than in paired cfDNA.

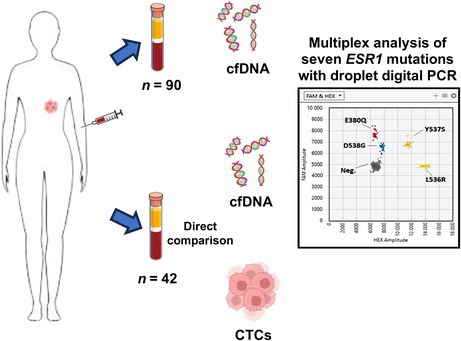

**Abbreviations**
AI: aromatase inhibitor
AKT1: AKT serine/threonine kinase 1
CDK4/6: cyclin‐dependent kinase 4 and 6
cfDNA: cell‐free DNA
CTCs: circulating tumor cells
ctDNA: circulating tumor DNA
ddPCR: droplet digital PCR
EDT: estrogen deprivation therapy
EDTA: ethylenediaminetetraacetic acid
EpCAM: epithelial cell adhesion molecule
ER: estrogen receptor
*ESR1*
: estrogen receptor 1
ET: endocrine therapy
EVs: extracellular vesicles
gDNA: genomic DNA
HD: healthy donors
IC: internal control
LB: liquid biopsy
LBD: ligand‐binding domain
MBC: metastatic breast cancer
NA: nucleic acid
PB: peripheral blood
PC: positive control
PD: progression of disease
PFS: progression‐free survival
PIK3CA: phosphatidylinositol‐4,5‐bisphosphate 3‐kinase catalytic subunit alpha
WT: wild‐type

## 1 Introduction

Endocrine therapy (ET) serves as the primary treatment for patients with metastatic breast cancer (MBC) who test positive for estrogen receptors (ER) [1]. Nevertheless, 40% of these patients do not experience any clinical benefit from initial ET, and almost all patients who initially respond to the treatment will eventually develop resistance [2]. Somatic mutations in the estrogen receptor 1 (*ESR1*) gene, particularly within the ligand‐binding domain (LBD) of the receptor (e.g., *ESR1* p.Y537N/C/S and p.D538G), promote constitutive and ligand‐independent activation of the ER, which impact 20–50% of MBC treated with aromatase inhibitors. The discovery of *ESR1* mutations has highlighted a significant mechanism likely responsible for acquired resistance to ET [3, 4].

Liquid biopsy (LB) is a minimally invasive tool that has been proven to be effective in monitoring treatment outcomes for various cancer types [5, 6]. LB techniques in cancer clinical diagnosis, therapy monitoring, and prognosis prediction encompass analysis of circulating tumor cells (CTCs), circulating tumor DNA (ctDNA), and extracellular vesicles (EVs) [7, 8]. In recent years, the advent of highly sensitive droplet digital PCR (ddPCR) assays has provided new opportunities for detecting mutations in ctDNA and CTC‐derived gDNA in cancer patients [9, 10]. Robust validation studies are crucial for demonstrating the clinical value and reliability of LB diagnostics in terms of validation and reproducibility [11].

In breast cancer (BC), an increase in the number of CTCs and an increase in the concentration of ctDNA have been shown to correlate with progression of disease (PD) [12, 13]. Tracking *ESR1* mutations through plasma‐cfDNA analysis is highly beneficial for the identification of tumor molecular dynamics and the improvement of personalized treatments for MBC patients as this is highlighted by several studies [14, 15, 16]. Although plasma‐cfDNA has become the most frequent analyte used to evaluate the mutational status of the tumor, CTCs are becoming a powerful alternative tool. A proof‐of‐concept study has already demonstrated the success of a liquid biopsy‐guided therapy by assessing *ESR1*, AKT serine/threonine kinase 1 (*AKT1*), and phosphatidylinositol‐4,5‐bisphosphate 3‐kinase catalytic subunit alpha (*PIK3CA*) mutations in the CTCs of four MBCs from serial blood draws [17]. Another study on *ESR1* mutation detection in single CTCs during estrogen deprivation therapy (EDT) demonstrated that CTC analysis could be an important tool to guide individualized therapy by identifying patients with a worse outcome under EDT [18], that may benefit from an early switch to an alternative ET or other treatment. Findings also confirm that the presence of an *ESR1* LBD mutation in CTCs or ctDNA is linked to a shorter progression‐free survival (PFS) when treated with an aromatase inhibitor (AI)‐based regimen [4]. A blood‐based test for *ESR1* mutation analysis would facilitate regular testing, allowing monitoring of the sensitivity to estrogen therapy and guiding treatment strategies [19].

The ASCO Expert Panel recently recommended routine testing for the emergence of *ESR1* mutations at recurrence or progression on ET, administered with or without cyclin‐dependent kinase 4 and 6 (CDK4/6) inhibitor, in patients with ER‐positive, HER2‐negative MBC [20]. Blood‐based ctDNA analysis is preferred due to its greater sensitivity in aiding treatment selection [21]. *ESR1* mutations can drive disease progression and may appear during first‐line treatment with ET [22]. Elacestrant is prescribed for managing advanced or MBC with disease progression in postmenopausal women or adult men who have ER‐positive, HER2‐negative, *ESR1*‐mutated tumors, and have already undergone at least one line of ET [23, 24, 25].

In the present study, we investigated whether analysis of *ESR1* mutations in CTCs and ctDNA provide complementary information on resistance to ET. Using a highly sensitive ddPLEX ddPCR assay, we detected a higher percentage of mutations in CTC‐derived gDNAs than in paired ctDNA in patients with MBC. Our results indicate that CTC‐derived gDNA analysis should be further evaluated as an important and complementary tool to ctDNA for identifying patients with *ESR1* mutations and for guiding individualized therapy.

## 2 Materials and methods

### 2.1 Sample collection and processing

Peripheral blood (PB) in ethylenediaminetetraacetic acid (EDTA) (10 mL) was collected from 90 patients with ER+ MBC, before the beginning of a new cycle of treatment, and 10 healthy donors (HD). PB samples were collected in participating clinical centers in the context of the Operational Program ‘Competitiveness, Entrepreneurship and Innovation’, under the call RESEARCH‐CREATE‐INNOVATE (project code: T1RCI‐02935). The study methodologies conformed to the standards set by the Declaration of Helsinki. All patients gave their informed written consent, and the study was approved by the Ethics committees of all participating institutions. PB samples were collected during the period of September 2019 to May 2022 from the University General Hospital of Alexandroupolis (720/03‐09‐2019), Alexandra Hospital (104/10‐02‐2021), Hygeia Hospital (36 627/23‐03‐2021), Metropolitan Hospital (2725/02‐12‐2019), Aretaieion University Hospital (173/30/1/2020), and Attikon University General Hospital (592/19‐09‐2019). Plasma was obtained from 90 patients by two consecutive centrifugations (530 ***g*** for 10 min at room temperature followed by a second centrifugation at 2000 ***g*** for 10 min) within 2–4 h and was further stored at −70 °C until analyzed. cfDNA was extracted from 2 mL of plasma using the QIAamp DSP circulating nucleic acid (NA) kit (Qiagen, Hilden, Germany), and cfDNA was eluted in 30 μL elution buffer, according to manufacturer's instructions [26]. CTC enrichment was performed in identical blood draws from 42 paired samples with epithelial cell adhesion molecule (EpCAM) positive immunomagnetic isolation receiving pooled CTCs, as previously described [27]. CTC‐derived gDNA was extracted using TRIzol™ LS as previously described [28]. DNA quantification in all samples was performed using the NanoDrop™ 1000 Spectrophotometer (Thermo Fisher Scientific, Waltham, MA, USA). All experimental procedures were performed in different lab rooms, with dedicated labware to avoid contamination. All preparation steps for the ddPCR setup were performed in a dedicated pre‐PCR room and a PCR hood dedicated for the preparation of ddPCR reactions.

### 2.2 ddPLEX *ESR1* mutation detection

**Fig. 1:**
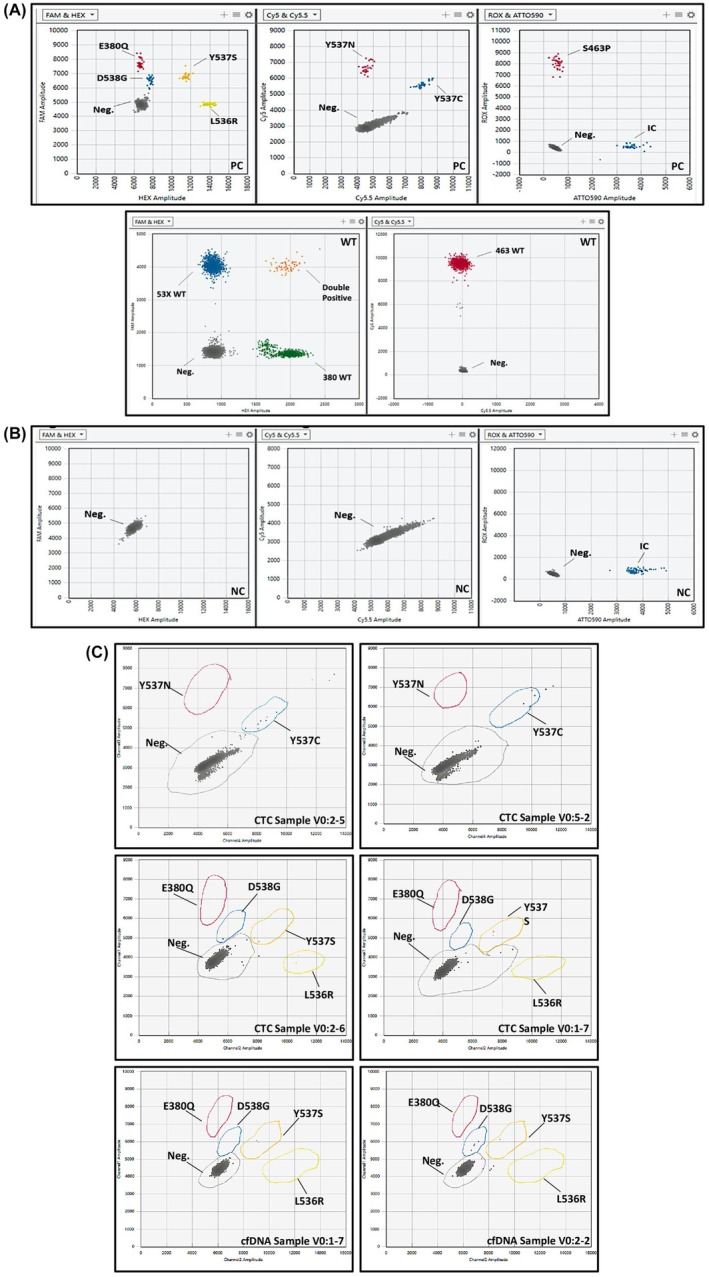
Representation of QX600™ Droplet Digital™ PCR System (Bio‐Rad, Hercules, CA, USA) 2D‐dot plot of the (A) positive control in ddPLEX *ESR1* assay and WT assay, (B) WT control and (C) *ESR1*‐positive samples. In positive control for *ESR1* assay all seven mutations and the Internal Control (IC) clusters are presented. In WT only the IC is presented. In 1C, samples V0:2‐5 and V0:5‐2 are *ESR1* p.Y537C, sample V0:2‐6 is *ESR1* p.Y537s, p.D538G, and p.L536R positive, and V0:1‐7 is *ESR1* p.Y537S‐positive in CTC‐derived gDNA. Samples V0:1‐7 and V0:2‐2 are *ESR1* p.Y537S and p.D538G positive in plasma‐cfDNA respectively.

The ddPLEX Mutation Detection Assay (Bio‐Rad) comprises two different assays. The wild‐type assay (WT Assay) measures the wild‐type loci in three exons of the *ESR1* gene (exons 5, 7, and 8). The Mutant Assay quantifies seven common variants (E380Q, S463P, L536R, Y537C, Y537N, Y537S, and D538G) and includes an internal control (IC). The ddPLEX *ESR1* assay also has a positive control (PC), that in addition to verifying the performance of the reaction serves as a guide for data thresholding, as it produces positive droplet clusters for each target (Fig. 1A). IC consists of a synthetic, nonhuman DNA sequence in solution and serves as an exogenous control that is introduced into the *ESR1* Mutant Assay reaction mixture for monitoring the efficacy of the ddPCR reaction (Fig. 1B). The kit is intended for use with the Bio‐Rad QX600™ Droplet Digital™ PCR System or QX600™ AutoDG Droplet Digital™ PCR System. The sample input into the ddPLEX *ESR1* Mutant assay was 5 μL and between 2 and 132 ng. An input of between 0.8 and 53 ng with a volume of 2 μL is recommended for the WT assay to conserve sample volume. The cycling conditions for *ESR1* Mutant and WT Assay are as following: enzyme activation 95 °C, 10 min, denaturation 95 °C, 10 s and annealing/extension 66 °C, 1 min for 37 cycles (ramp rate 2 °C·s^−1^), enzyme deactivation 98 °C, 10 min and hold at 4 °C for at least 30 min or until the lid temperature is 37 °C or lower.

## 3 Results

**Fig. 2:**
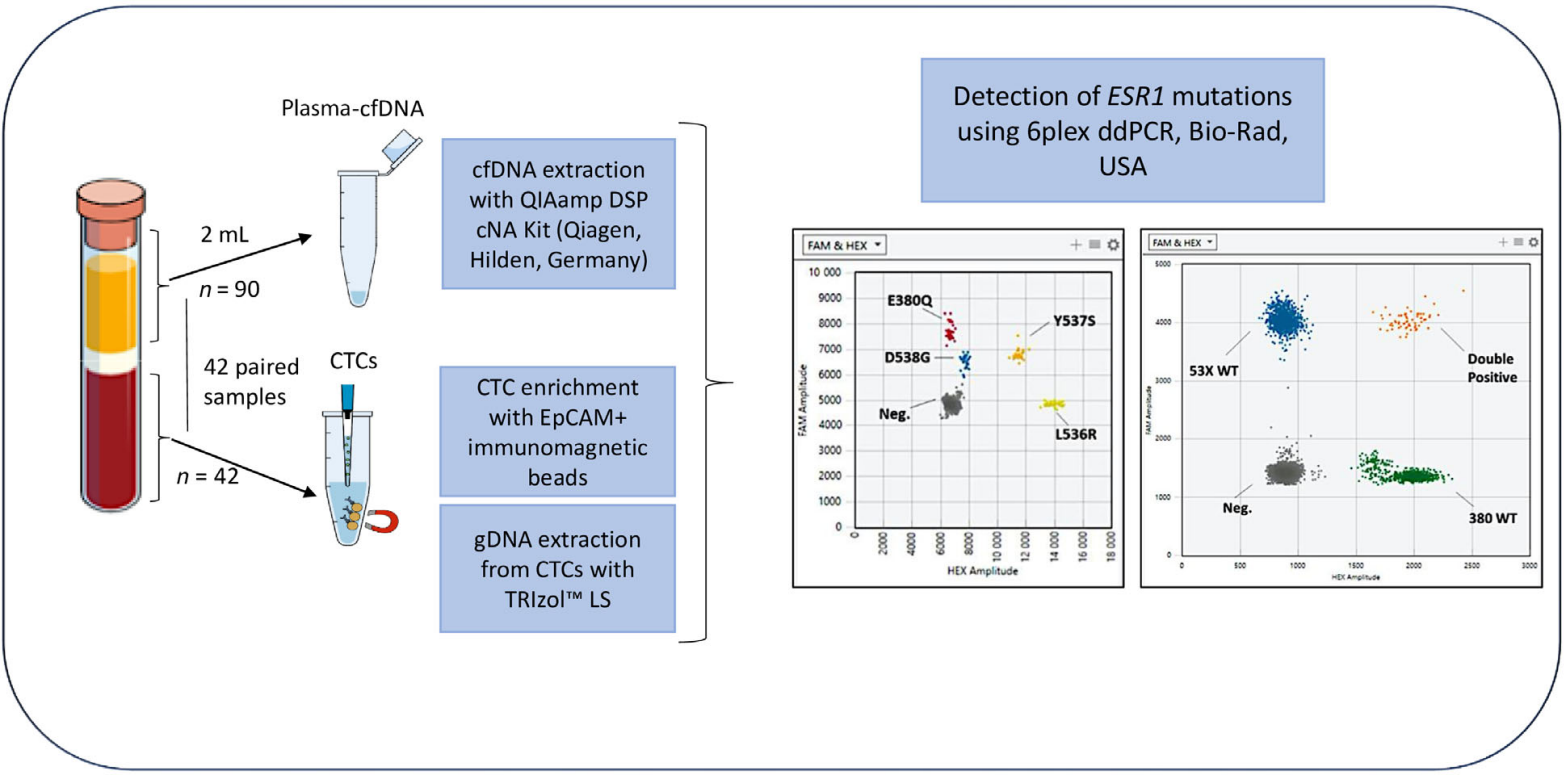
Experimental workflow of the study. Left: Isolation of plasma‐cfDNA (*n =* 90) and paired CTC‐derived gDNA (*n =* 42). Right: Detection of *ESR1* mutations using ddPLEX Mutation Detection Assay. cfDNA, cell‐free DNA; CTC, circulating tumor cell; gDNA, genomic DNA; LS, lysis solution.

A detailed experimental flowchart of the study is presented in Fig. 2.

### 3.1 Detection of *ESR1* mutations in plasma‐cfDNA

**Fig. 3:**
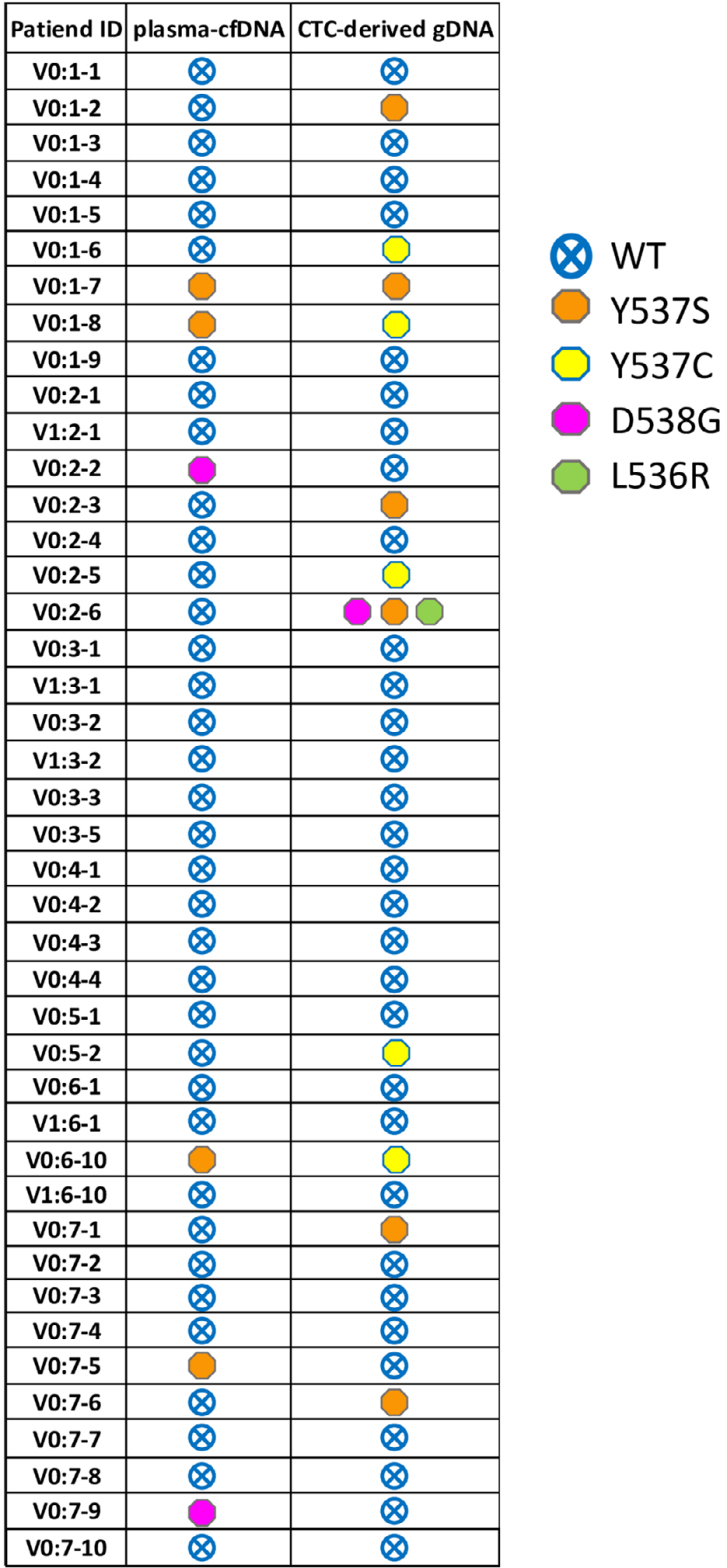
Direct comparison of *ESR1* mutations detected in plasma‐cfDNA and paired CTC‐derived gDNAs for 42 samples. Each *ESR1* mutation or wild‐type (WT) is presented with a different symbol. *ESR1* mutations are more frequent in CTC‐derived gDNAs. CTC, circulating tumor cell; gDNA, genomic DNA.

The ddPLEX Mutation Detection Assay is highly specific, since all plasma‐cfDNA samples from HD (*n =* 10) were found negative for all seven *ESR1* mutations tested. For quality control reasons, in each sample an exogenous IC was added and quantified and the accepted concentration should range from 3.0 to 5.5 copies·μL^−1^. Using this test, eight out of 90 (8.9%) plasma‐cfDNA samples of MBC patients were found to be positive for one of these seven *ESR1* mutations (Fig. 3). Six patients (Pt#V0:1‐7, Pt#V0:1‐8, Pt#V0:6‐10, Pt#V0:7‐5, Pt#V0:8‐1, and Pt#V0:8‐2) were found positive for the *ESR1* p.Y537S mutation, and two patients (Pt#V0:2‐2, and Pt#V0:7‐9) were found positive for the *ESR1* p.D538G mutation. Mutant allele frequency (MAF%) for *ESR1* p.Y537S mutation in plasma‐cfDNA ranged from 0.15% to 9.51%, while for *ESR1* p.D538G MAF% was found 0.20% and 0.64% (Table 1).

**Table 1:** Mutation analysis results of *ESR1*‐positive samples in plasma‐cfDNA and paired CTC‐derived gDNAs.

Patient ID	LB analyte	Mutation, copies·μL^−1^	WT assay, copies·μL^−1^	Mutant allele frequency (MAF)
V0:1‐2	cfDNA	Non‐mutated	Non‐mutated	Non‐mutated
	CTC	123.64	333.74	4.35%
V0:1‐6	cfDNA	Non‐mutated	Non‐mutated	Non‐mutated
	CTC	11.5	4740.96	2.42%
V0:1‐7	cfDNA	0.54	204.6	**0.26%**
	CTC	0.70	694.87	**0.10%**
V0:1‐8	cfDNA	0.82	63.8	1.27%
	CTC	0.57	1846.24	0.03%
V0:2‐2	cfDNA	1.1	537.9	0.20%
	CTC	Non‐mutated	Non‐mutated	Non‐mutated
V0:2‐3	cfDNA	Non‐mutated	Non‐mutated	Non‐mutated
	CTC	16.8	724.44	2.27%
V0:2‐5	cfDNA	Non‐mutated	Non‐mutated	Non‐mutated
	CTC	52.6	1295.8	3.90%
V0:2‐6	cfDNA	Non‐mutated	Non‐mutated	Non‐mutated
	CTC	12.2	1946.95	0.62%
V0:5‐2	cfDNA	Non‐mutated	Non‐mutated	Non‐mutated
	CTC	1.0	936.47	0.11%
V0:6‐10	cfDNA	2.7	31.02	8.01%
	CTC	50.4	703.69	6.68%
V0:7‐1	cfDNA	Non‐mutated	Non‐mutated	Non‐mutated
	CTC	33.9	814.45	3.99%
V0:7‐5	cfDNA	117.7	1119.8	9.51%
	CTC	Non‐mutated	Non‐mutated	Non‐mutated
V0:7‐6	cfDNA	Non‐mutated	Non‐mutated	Non‐mutated
	CTC	2.5	1172.49	0.21%
V0:7‐9	cfDNA	1.0	156.2	0.64%
	CTC	Non‐mutated	Non‐mutated	Non‐mutated
V0:8‐1	cfDNA	0.52	353.65	0.15%
V0:8‐2	cfDNA	0.51	96.8	0.52%

### 3.2 Detection of *ESR1* mutations in paired CTC‐derived gDNAs

A total number of 42 paired CTC‐derived gDNA samples were analyzed for the detection of *ESR1* mutations using the ddPLEX Mutation Detection Assay. *ESR1* mutations in samples were demonstrated in different clusters in a 2D‐plot (Fig. 1A). Eleven out of 42 (26.2%) CTC‐derived gDNA samples were found positive for at least one *ESR1* mutation (Fig. 3). More specifically, the *ESR1* p.Y537S was detected in six (Pt#V0:1‐2, Pt#V0:1‐7, Pt#V0:2‐3, Pt#V0:2‐6, Pt#V0:7‐1, and Pt#V0:7‐6) CTC‐derived gDNAs and the *ESR1* p.Y537C in five (Pt#0:1‐6, Pt#V0:1‐8, Pt#V0:2‐5, Pt#V0:5‐2, and Pt#V0:6‐10). It should be mentioned that in one sample (Pt#V0:2‐6) the *ESR1* p.Y537S was detected in parallel with the *ESR1* p.D538G and the *ESR1* p.L536R. The MAF% for *ESR1* p.Y537S varied from 0.1% to 4.4% and for *ESR1* p.Y537C from 0.03% to 6.7% (Table 1). For the sample of Pt#V0:2‐6, all three *ESR1* mutations (p.Y537S, p.D538G, and p.L536R) were detected with the same MAF% (0.62%). It should be highlighted that the MAF of 0.03% which was detected in sample of Pt#V0:1‐8 for the *ESR1* p.Y537C mutation, was very low, but it was above the MAF (%) received from WT control, and thus, this sample was accepted as *ESR1* p.Y537C positive.

### 3.3 Direct comparison of *ESR1* mutations in paired plasma‐cfDNA and CTC‐derived gDNAs

Direct comparison of paired plasma‐cfDNA and CTC‐derived gDNA samples (*n =* 42) revealed that the detection rate for *ESR1* mutations was higher in CTC‐derived gDNA (11/42, 26.2%) than in plasma‐cfDNA samples (6/42, 14.3%) (Table S1). In three patients (Pt#V0:1‐7, Pt#V0:1‐8, and Pt#V0:6‐10), one *ESR1* mutation was detected in both plasma‐cfDNA and paired CTC‐derived gDNA (Table 2). Only Pt#V0:1‐7 had the *ESR1* p.Y537S mutation in both LB analytes (Fig. 1C). Eight more patients (Pt#V0:1‐2, Pt#V0:1‐6, Pt#V0:2‐3, Pt#V0:2‐5, Pt#V0:2‐6, Pt#V0:5‐2, Pt#V0:7‐1, and Pt#V0:7‐6) were found positive for *ESR1* mutations in CTC‐derived gDNA [Concordance: 31/42 (73.81%), *P* < 0.152, chi‐squared test] (Table 2). Two patients (Pt#V0:1‐8 and Pt#V0:6‐10) were found positive for *ESR1* p.Y537S mutation in plasma‐cfDNA, while one additional mutation, *ESR1* p.Y537C, was detected only in CTCs (Fig. 3). Taken together, CTC and ctDNA genotyping give complementary information on the presence of *ESR1* mutations in patients' samples.

**Table 2:** Direct Comparison of *ESR1* mutations in paired plasma‐cfDNA and CTC‐derived gDNAs.

	CTC‐derived gDNA			
Plasma‐cfDNA		+	−	Total
	+	3	3	6
	−	8	28	36
	Total	11	31	42
	Concordance: 31/42 (73.81%), *p* < 0.152, chi‐squared test			

### 3.4 Treatment regimens for patients positive for *ESR1* mutations

**Fig. 4:**
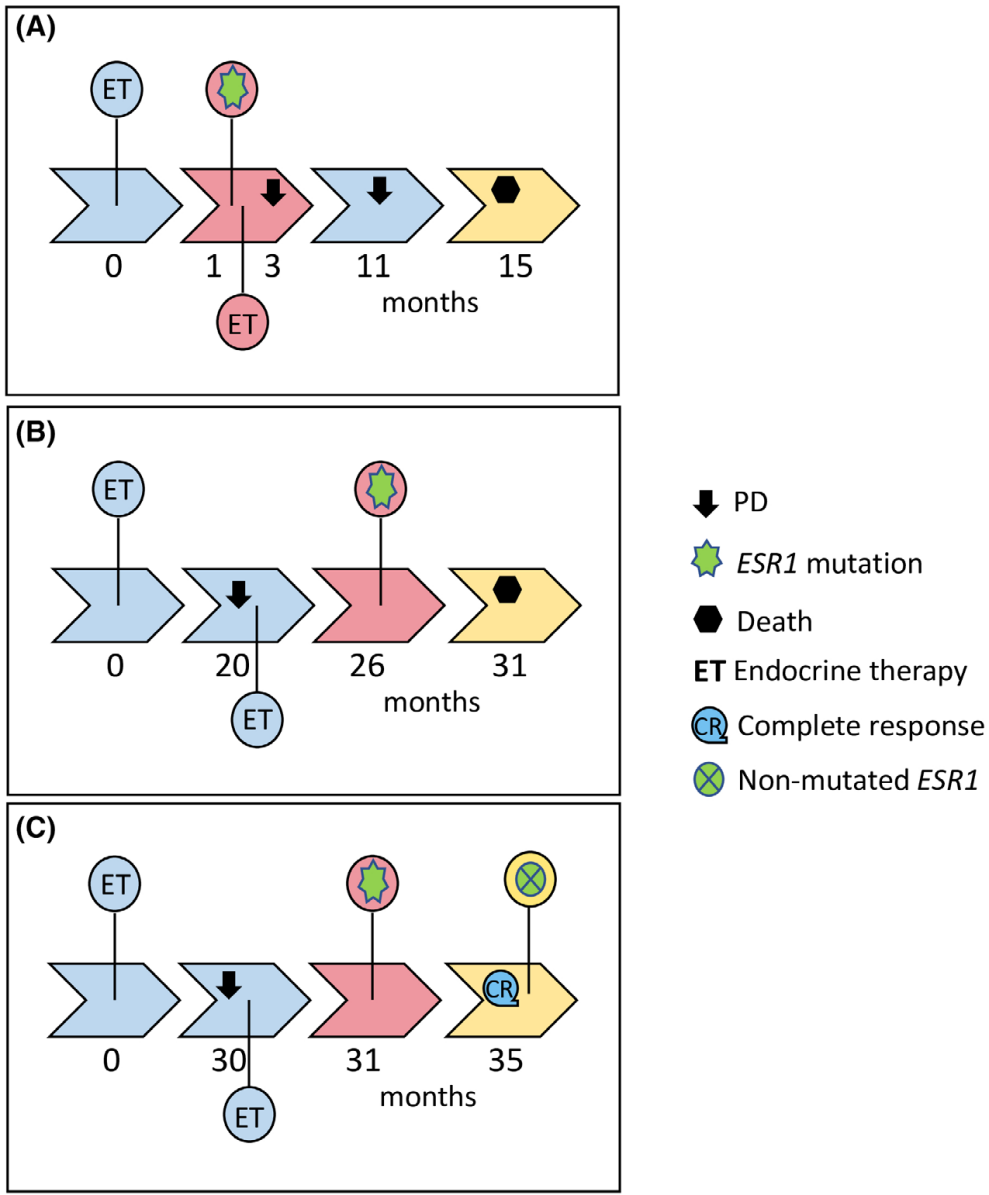
Graphical illustration of disease progression for three different patients. (A) Pt#V0:2‐2 received endocrine therapy (ET) for 15 months until the date of blood sampling, then switched to chemotherapy plus immunotherapy, and 2 months later had progression of disease (PD). At this time point, *ESR1* p.D538G was detected in plasma‐cfDNA. However, according to the clinician, the patient kept receiving chemotherapy plus immunotherapy, and after 4 months from new PD and resistance to therapy, the patient passed away. (B) Pt#V0:2‐3, who received ET as a first‐line treatment, 20 months later progressed. After 1 month from relapse the patient received again ET, without knowing the *ESR1* mutation status, and 10 months later passed away. *ESR1* mutations were not detected in plasma‐cfDNA of this patient, and however, 6 months after PD *ESR1* p.Y537S was detected in CTC‐derived gDNA. (C) Pt#V0:6‐10 had PD already before blood sampling. This patient received ET as first‐line treatment already and she finally progressed 30 months after diagnosis. One month after the relapse, *ESR1* p.Y537S and *ESR1* p.Y537C were detected in plasma‐cfDNA and in CTC‐derived gDNA, respectively, and 4 months later none of these mutations were detected neither in plasma‐cfDNA nor in CTC‐derived gDNA. The patient had complete response to treatment.

From eight patients who were positive for *ESR1* mutation in plasma‐cfDNA, five (Pt#V0:1‐7, Pt#V:1‐8, Pt#V0:7‐9, Pt#V0:8‐1, and Pt#V0:8‐2) received chemotherapy plus ET and two patients (Pt#V0:6‐10 and Pt#V0:2‐2) received only ET before the blood draw. Pt#V0:2‐2 received ET for 15 months until the date of blood sampling (Fig. 4A), then switched to chemotherapy plus immunotherapy, and 2 months later had PD. At this time point, *ESR1* p.D538G was detected in plasma‐cfDNA. However, according to the clinician, the patient kept receiving chemotherapy plus immunotherapy, and after 4 months from new PD and resistance to therapy, the patient passed away. The invaluable information for the presence of *ESR1* mutation in ctDNA before the second line of therapy could have been of benefit for the patient by switching on time to another more efficient treatment.

Furthermore, it was observed that five MBC patients (5/11, 45.5%) with *ESR1* mutations detected in their CTC‐derived gDNAs had PD before blood sampling. Pt#V0:2‐3, who received ET as a first‐line treatment, 20 months later progressed (Fig. 4B). After 1 month from relapse, the patient received again ET, without knowing the *ESR1* mutation status, and 10 months later passed away. *ESR1* mutations were not detected in plasma‐cfDNA of this patient, and however, 6 months after PD *ESR1* p.Y537S was detected in CTC‐derived gDNA. The information for the presence of *ESR1* mutation in CTC‐derived gDNA in the beginning of the second‐line ET could have been of benefit for the patient if she would have been offered another treatment.

From the three MBC patients (Pt#V0:1‐7, Pt#V0:1‐8, and Pt#V0:6‐10) that were found positive for *ESR1* mutations in both LB analytes (plasma‐cfDNA and CTC‐derived gDNA), one patient (Pt#V0:6‐10) had PD already before blood sampling. This patient received ET as first‐line treatment already and she finally progressed 30 months after diagnosis (Fig. 4C). One month after the relapse, *ESR1* p.Y537S and *ESR1* p.Y537C were detected in plasma‐cfDNA and in CTC‐derived gDNA, respectively. The patient continued to receive ET plus immunotherapy and 4 months later none of these mutations were detected either in plasma‐cfDNA or in CTC‐derived gDNA. The patient had complete response to treatment with denosumab.

## 4 Discussion

In the present study, we report the detection of seven *ESR1* mutations (p.E380Q, p.S463P, p.L536R, p.Y537C, p.Y537N, p.Y537S, and p.D538G) simultaneously in plasma‐cfDNA and paired CTC‐derived gDNA samples from MBC patients using a highly sensitive and specific ddPLEX Mutation Detection Assay. A WT Assay for the measurement of the WT loci in three exons of the *ESR1* gene (exons 5, 7, and 8) was also used as part of the same assay. Our results indicate that the detection rate for *ESR1* mutations was significantly higher in CTC‐derived gDNA than in paired plasma‐cfDNA samples.

In a previous study design, it was reported that harboring an *ESR1* LBD mutation in CTCs or ctDNA was linked to a shorter PFS when undergoing treatment with an AI‐based regimen. This finding suggests that a CTC blood‐based assay could serve as an important and complementary tool to ctDNA for identifying patients with *ESR1* mutations and for guiding individualized therapy, and it aligns with comparable data from plasma DNA‐based mutation analysis [4]. The findings of the mutation analysis (*PIK3CA*, *ESR1*, *TP53*, and *KRAS*) of cfDNA and single CTCs in MBC patients with high CTC counts indicate that when considering the use of EpCAM‐positive CTCs, it may be crucial to simultaneously analyze serial samples of CTCs for making clinical decisions, especially in cases where a metastatic biopsy cannot be performed [29]. Similarly, our results indicate that in cases that *ESR1* mutations were not detected in plasma‐cfDNA then the presence of *ESR1* mutations in EpCAM‐positive CTCs in patients could facilitate the monitoring of metastatic burden for making clinical decisions.

Elacestrant has recently been demonstrated to significantly extend PFS compared to standard endocrine monotherapy in Phase III EMERALD study [23, 24]. This improvement was observed in both *ESR1* mutant and *ESR1* WT patients. However, the hazard ratio revealed a greater PFS prolongation effect from elacestrant in *ESR1* mutant patients compared to all patients, regardless of their *ESR1* status. In the *ESR1*‐mutated population, PFS was improved in those who had been exposed to CDK4/6 inhibitors for a longer period [23].

In the case of MBC, analyzing plasma‐cfDNA can help identify specific genetic mutations that are suitable for targeted therapy. There are several approved tests available to detect genetic mutations in plasma‐cfDNA, which can assist in choosing the most appropriate treatment [30]. For example, *ESR1* mutations can be identified for the selection of treatment with the estrogen receptor‐targeted therapy elacestrant. *ESR1* mutations are often polyclonal, and longitudinal analysis reveals distinct clones with varying behavior over time [31]. *In vitro* studies found that constitutively active mutants p.Y537S and p.D538G are less sensitive to fulvestrant, suggesting potential clinical resistance of *ESR1* mutant patients to achievable fulvestrant exposures [32, 33]. Based on our findings, CTCs provide valuable information about the tumor's *ESR1* mutation status. Therefore, MBC patients with *ESR1*‐mutated CTCs may potentially benefit from elacestrant, regardless of the *ESR1* mutation status in ctDNA.

The main finding of this pilot technical study, that *ESR1* mutations are detected at a higher percentage in CTCs than in ctDNA could be very important for selecting the appropriate therapy for these patients. One limitation of this study is the small sample size; we believe that this is the main reason that this difference in percentages is not clearly statistically significant but there is an obvious trend. Even if we started with a larger number based on the quality control of CTC‐derived gDNA, we could only perform direct comparison of CTCs and ctDNA in 42 patient samples, using the same blood draws and the same downstream analysis technology. Ideally, our findings should be further evaluated in a well‐designed clinical study involving more patients and samples derived at the same time points, that will also distinct stochastic variation in detecting *ESR1* mutations in CTCs and/or cfDNA from biological variation.

## 5 Conclusions

In conclusion, the findings from our study strongly indicate that the detection of *ESR1* mutations could be beneficial for patients with MBC and complementary information for *ESR1* status of plasma‐cfDNA and CTC‐derived gDNA could improve therapy selection. CTC‐derived gDNA analysis should be further evaluated as an important and complementary tool to ctDNA analysis for identifying patients with *ESR1* mutations and for guiding individualized therapy.

## Conflict of interest

The authors declare no conflict of interest.

## Author contributions

SS, AN, and VT carried out the experiments; IB, AP, FZ, HL, ER, SK, APs, and CP provided the clinical samples used in this study; SS and EL took the lead in writing the manuscript; EL provided critical feedback and helped shape the research, analysis, and manuscript. All authors have read and agreed to the published version of the manuscript.

## Peer review

The peer review history for this article is available at https://www.webofscience.com/api/gateway/wos/peer‐review/10.1002/1878‐0261.13787.

## Supporting information


**Table S1.** *ESR1* mutation status of 42 plasma‐cfDNA and paired CTC‐derived gDNA samples.

## Data Availability

All data generated or analyzed during this study are included in this publication and/or are available from the corresponding author upon reasonable request.
