# Supplementary figures and images for: Detection rate for ESR1 mutations is higher in circulating‐tumor‐cell‐derived genomic DNA than in paired plasma cell‐free DNA samples as revealed by ddPCR
Source: Mol Oncol. 2025 Jan 4;19(7):2109–19. doi: 10.1002/1878-0261.13787 (PMC12234389; doi:10.1002/1878-0261.13787)

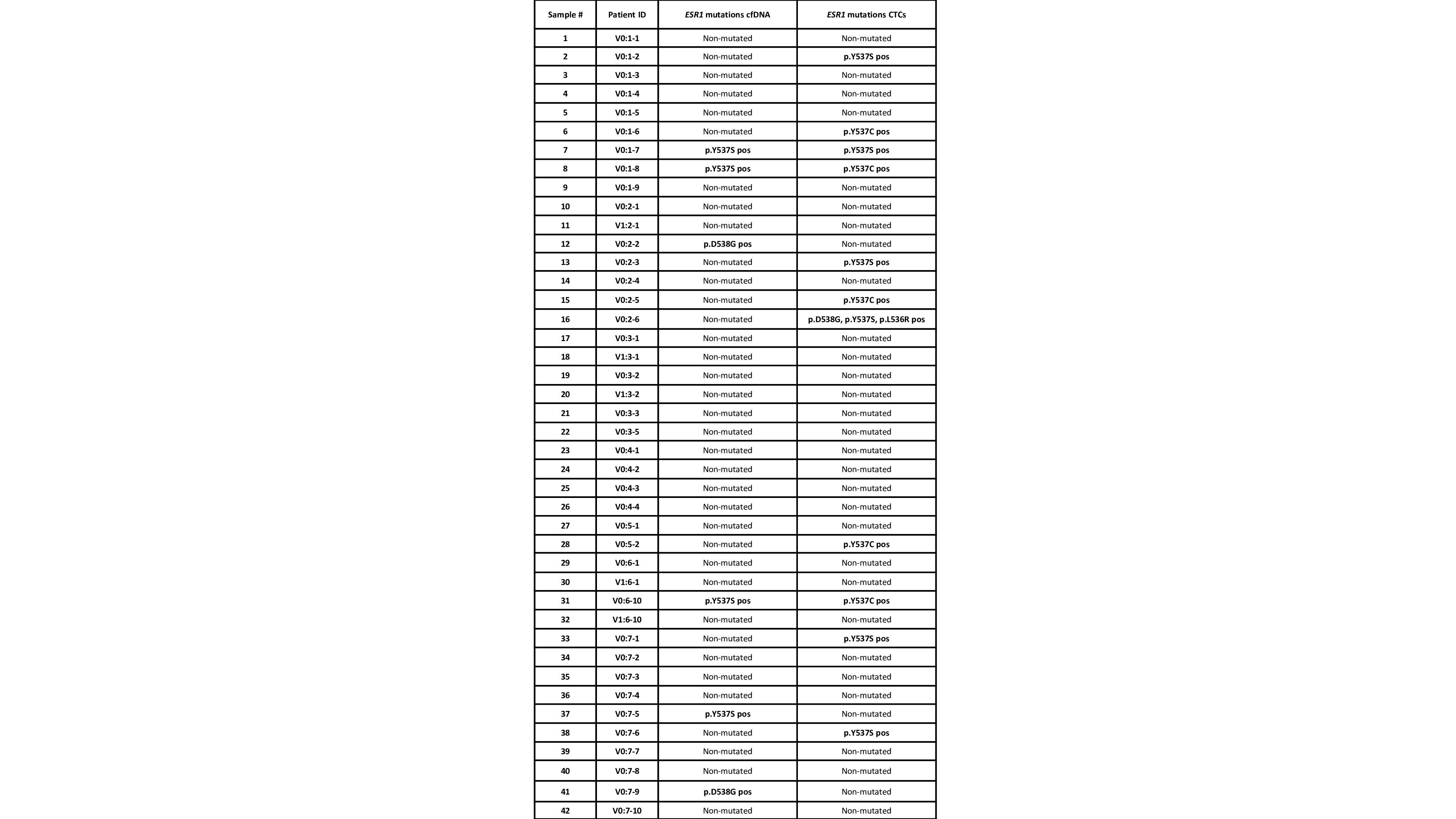

Supplement: Supplementary file 1 — Table S1. ESR1 mutation status of 42 plasma‐cfDNA and paired CTC‐derived gDNA samples. [file MOL2-19-2109-s001.jpeg]
